# The lived experience of intimate partner violence among first-generation latino immigrants in Southern New Mexico

**DOI:** 10.1371/journal.pone.0361485

**Published:** 2026-10-27

**Authors:** Jochebed Ade-Oshifogun, Jodi Patterson, Kwabena Adu Agyemang, Angelica Carabajal

**Affiliations:** 1 School of Nursing, College of Health, Education and Social Transformation, New Mexico State University, Las Cruces, New Mexico, United States of America; 2 Department of Computer Science, College of Arts and Social Sciences, New Mexico State University, Las Cruces, New Mexico, United States of America; Independent Global Health Consultant, INDIA

## Abstract

Intimate partner violence (IPV) is a significant public health concern that disproportionately affects immigrant populations. First-generation Latino immigrants in the United States experience IPV within complex cultural, relational, and structural contexts that remain underexplored. This study aimed to explore and describe how first-generation Latino immigrants in Southern New Mexico experience and make meaning of IPV and to examine the cultural and relational influences shaping these experiences. A qualitative phenomenological design was used, and reporting followed the Consolidated Criteria for Reporting Qualitative Research (COREQ) guidelines. Twenty-four first-generation Latino immigrants were interviewed; two who described observing rather than personally experiencing IPV were excluded, yielding an analytic sample of 22 who participated in in-depth, semi-structured interviews conducted in English or Spanish using a trauma-informed and culturally responsive approach. Interviews were audio-recorded, transcribed verbatim, and translated where necessary. Data was analyzed using thematic analysis informed by phenomenological principles, supported by Intellectus® Qualitative software and manual coding to ensure rigor and depth. Data saturation was reached during data collection. Six major themes emerged: cultural factors, abuse, culture of silence, control, universal factors, and systemic barriers to legal and support resources. Participants described IPV as shaped by cultural norms, normalization of violence, fear, dependency, and limited access to resources. A pervasive culture of silence, reinforced by stigma, immigration-related fears, and social isolation, constrained help-seeking behaviors. Structural barriers further limited access to legal protections and support systems, exacerbating vulnerability. Findings highlight the need for culturally responsive, multilevel interventions that address the social, cultural, and structural determinants of IPV among Latino immigrant populations. Efforts to improve access to support services and reduce systemic barriers are critical to enhancing safety and positive health outcomes.

## Introduction

Intimate partner violence (IPV) is a major global public health problem and a violation of human rights. The World Health Organization estimates that nearly one in three women worldwide have experienced physical and/or sexual violence by an intimate partner, or non-partner sexual violence, or both during their lifetime [1]. Intimate partner violence poses significant public health concerns with lifelong impacts on the health and well-being of individuals [2], families, and communities worldwide [1]. IPV can have fatal outcomes, as approximately one in five homicide victims in the United States are killed by a current or former intimate partner [2]. In addition to its human toll, IPV imposes a substantial societal burden, with estimated lifetime economic costs exceeding $3.6 trillion due to healthcare expenditures, lost productivity, criminal justice involvement, and other related costs [2].

Consistent with WHO findings, one in three adults in New Mexico experience IPV at least once in their lifetime [3]. Doña Ana County accounts for approximately 24% of reported domestic violence cases, representing one of the highest proportions in the state [4]. Among reported survivors, the majority are female (93%) and Hispanic (56%), reflecting both the gendered nature of IPV and the demographic composition of the region. These patterns highlight the urgent need for culturally responsive approaches to IPV assessment, screening, and prevention in southern New Mexico, where Latino immigrant communities are strongly represented [4].

The purpose of this qualitative study is to explore the lived experiences of intimate partner violence (IPV) among first-generation Latino immigrants and to examine the cultural and contextual factors that shape those experiences. By centering the perspectives of survivors residing in Southern New Mexico, this study seeks to generate an in-depth understanding of IPV as experienced within this community and to inform the future development of a culturally sensitive risk assessment questionnaire.

The study pursued two objectives: to explore and describe how first-generation Latino immigrants in Southern New Mexico experience and make meaning of intimate partner violence and to examine the contextual, cultural, and relational influences shaping these experiences as revealed through thematic analysis of in-depth interviews.

### Literature review

This literature review examines the evidence related to IPV risks and outcomes, IPV and the Latino population, and existing IPV assessment tools.

### IPV

#### Risks.

Risks can be assessed at the societal, community, interpersonal, and individual levels. Societal risks include discriminatory laws on marriage, divorce, child custody, women’s employment [5], as well as cultural norms that support violence [2]. Increased risks at the community level include poverty, unemployment, violent crime, drugs, alcohol, and access to weapons, along with male privilege, lack of female autonomy, stigma [5], and lack of community engagement [2]. Interpersonal risks include relationships with inequality, infidelity/lack of monogamy, male drug/alcohol abuse [5], lack of education, economic stress, and witnessing and/or experiencing violence in youth [2]. Finally, individual risks include low self-esteem, young age, substance abuse, isolation, emotional dependence/insecurity [2], mental health disorders, and attitudes that normalize violence [5].

#### Outcomes.

Health outcomes associated with IPV include increased risk of the following: engaging in high-risk behaviors, contracting sexually transmitted infections, developing diabetes, chronic diseases, and/or pain, and adverse symptoms of menopause [6]. Mental health outcomes related to IPV include increased risks for depression, anxiety, posttraumatic stress disorder (PTSD), psychological distress, and suicidal ideations [7].

#### IPV and the Latina Population.

While IPV affects all racial and ethnic groups worldwide [1], Latina women experience unique vulnerabilities that are often insufficiently addressed in IPV research. The prevalence of IPV in this population is difficult to determine with varying estimates related to data collection methods, immigration policies, and variations in language used [8]. Latina survivors of IPV often experience more severe physical and mental health consequences relative to non-Latina survivor groups [9]. These disparities highlight the need to examine not only the prevalence of IPV but also the contextual factors that shape its impact within Latina populations.

#### Family and cultural values.

When discussing IPV in the Latina population, it is important to understand cultural constructs such as machismo, marianismo, and familismo. Machismo is conceptualized within patriarchal masculinity norms that emphasize male dominance, authority, and control, which may reinforce attitudes supportive of coercion and IPV [4,10,11]. Conversely, marianismo contains five pillars: family, virtue and chastity, subordination to others, self-silencing to maintain harmony, and spirituality [12]. Marianismo, then, prescribes submissive, self-sacrificing behavior for women, encouraging silence and endurance in the face of abuse [12]. These cultural scripts create environments where violence may be normalized or internalized by both partners.

In conjunction with machismo and marianismo, familismo emphasizes strong family loyalty, interdependence, and the prioritization of family unity over individual needs [13]. While familismo can serve as a protective factor by promoting social support and family cohesion, it may also create pressures that discourage women from disclosing abuse or leaving abusive relationships [14–16]. Within many Latino cultural contexts, women may feel responsible for preserving family harmony and avoiding actions that could disrupt family stability, which may lead some survivors to remain in abusive relationships or seek assistance only from trusted relatives rather than formal support services [17].

Da Silva et al. [18] emphasize that marianismo beliefs, particularly those promoting submissiveness and self-silencing, are strongly associated with Latina women’s psychological distress and may prevent them from recognizing or reporting IPV. Similarly, Sabina et al. [19] reported that traditional gender role expectations in some Latino communities may reinforce male authority and female self-sacrifice, contributing to relationship power imbalances associated with intimate partner violence.

Cultural values emphasizing respect, harmony, and avoidance of public conflict may contribute to perceptions that IPV should be managed within the family rather than disclosed externally [20,21]. Additionally, qualitative research with immigrant Latina survivors has shown that concerns about family reputation, community judgment, and the desire to maintain family unity can lead women to minimize abuse or delay formal intervention [16,22].

Finally, Catholicism remains the predominant religion among U.S. Latinos and often informs beliefs about marriage, family unity, gender roles, forgiveness, and relational commitment [23].

#### Immigration issues.

Immigration to the United States compounds IPV as it often brings fear, financial constraints, and multi-level knowledge deficits.

#### Fear.

Migration-related experiences may further complicate these dynamics. Acculturative stress, language barriers, limited familiarity with available services, fear related to immigration status, and concerns about deportation or family separation may all shape survivors’ responses to IPV [21,24,25]. Fears may be amplified by anti-immigration climates, uncertainty about legal protections, and misinformation about eligibility for services or support from law enforcement [24,26,27]. In some cases, abusive partners may exploit immigration-related fears by threatening deportation, withholding immigration documents, or using documentation status as a mechanism of control [10,21,24].

#### Financial constraints.

Socioeconomic factors such as financial dependency, unstable employment, limited educational opportunities, and restricted access to material resources may substantially increase vulnerability to intimate partner violence (IPV) and constrain help-seeking among Latina immigrant survivors [10,12]. Also, women experiencing IPV may face limited work opportunities due to childcare responsibilities, lack of transportation, language barriers, documentation concerns, and restricted access to community resources [12,28]. In some cases, abusive partners may actively interfere with survivors’ ability to achieve or maintain employment by restricting access to transportation, withholding identification or immigration documents, creating debt, controlling household finances, or threatening violence if the survivor attempts to work or become financially independent [12,28]. Alvarez et al. [13] found that some male partners responded with aggression when confronted with economic disempowerment, cultural dislocation, or perceived threats to traditional gender hierarchies.

#### Knowledge deficits.

Limited formal education, reduced health literacy, and difficulty communicating in English may restrict awareness of legal protections, employment options, and available support services [29]. Latina immigrant survivors may encounter substantial barriers when interacting with police, healthcare personnel, legal systems, or court processes, particularly when language discordance, lack of interpretation services, or limited understanding of procedural requirements are present [24,30,31].

#### Existing IPV assessment tools.

A few instruments have been developed to assess IPV, severity of abuse, and risk for future harm but may not fully capture the culturally and structurally specific vulnerabilities experienced by Latino immigrant populations. The Danger Assessment (DA) and related adaptations, (Danger Assessment for Immigrant Women [DA-I]) include domains such as social isolation, immigration-related stressors, and relationship power dynamics, but are limited in reflection of the lived experiences, cultural meanings, and regional realities of specific Latino immigrant communities [32]. Additionally, the Spanish version of the Index of Spouse Abuse (ISA) and brief screening items such as “¿Ha sentido sola?” have demonstrated utility in identifying abuse among Spanish-speaking women [8,33]. However, some of these measures are either too lengthy for routine clinical or community-based use or too narrow in scope to assess the broader sociocultural and structural dimensions of IPV risk. Also, the Culturagram may provide useful qualitative insight into family roles, migration history, and social context, but they are not standardized IPV risk measures and often require specialized training for consistent use [34].

These limitations are particularly relevant for Latino immigrant populations in Southern New Mexico, whose experiences of IPV may be shaped by intersecting challenges such as undocumented or mixed-status households, limited English proficiency, financial dependency, social isolation, fear of deportation, and reduced trust in formal systems [12,21,24]. Such factors may influence not only risk for abuse, but also whether survivors recognize, disclose, or seek help for IPV. When assessment tools do not explicitly account for these realities, there is a risk that abuse may be under identified, mischaracterized, or insufficiently contextualized in practice settings. Given the above limitations and the pressing need for culturally responsive care, developing a region-specific IPV risk assessment tool for Latino immigrants in southern New Mexico is not only justified, but also necessary. This research aims to study this population's experiences of IPV to identify themes that will inform future development of risk assessment tool items.

In summary, the reviewed literature underscores the importance of understanding intimate partner violence within culturally specific frameworks. The broader literature on intimate partner violence establishes IPV as a multifaceted phenomenon encompassing physical, emotional, psychological, and economic abuse, while studies focused on Latino immigrants emphasize unique risk factors linked to migration, acculturation, economic dependency, and legal vulnerability. Research examining the intersection of IPV and immigration further demonstrates how immigration-related stressors and systemic barriers can exacerbate power imbalances, limit help-seeking, and entrap survivors within abusive relationships. Collectively, this body of literature highlights critical gaps in culturally grounded research and assessment tools. These gaps provide a strong rationale for the present study, which seeks to explore the lived experiences of IPV among first-generation Latino immigrants in Southern New Mexico to inform culturally responsive understanding and future tool development efforts.

#### Theoretical framework.

The qualitative component of this study is guided by the Social Ecological Model (SEM) which frames intimate partner violence (IPV) as a lived experience shaped by interacting personal, relational, community, and societal contexts within the migration experience [2,19,26,35]. This framework allows for an in-depth exploration of how IPV is experienced, understood, and navigated by first-generation Latino immigrants in Southern New Mexico.

The SEM provides a useful structure for understanding IPV beyond individual behavior. At the individual level, SEM supports exploration of survivors’ personal histories, cultural beliefs, emotional responses, and prior experiences that influence how violence is perceived and endured. For immigrant survivors, these experiences are often intertwined with migration-related stressors such as economic insecurity, language barriers, and emotional strain associated with resettlement [12,24]. At the relationship level, SEM focuses on dynamics within intimate partnerships, including power imbalances, dependency, jealousy, communication patterns, and changes in roles following migration [19]. Shifts in gender expectations and differences in acculturation between partners may alter relationship dynamics and increase vulnerability to control or abuse. Qualitative research among Latina and immigrant women highlights how these relational processes shape the lived experience of IPV and influence survivors’ responses to violence [19,36].

The community level highlights the role of social environments, including family networks, cultural communities, and access to support services. For many Latina immigrants, community contexts may simultaneously offer support and impose constraints. Social isolation, fear of stigma, and limited access to culturally responsive services can restrict help-seeking and reinforce silence around IPV [19,37]. At the societal level, SEM draws attention to broader structural forces that shape IPV experiences, including immigration policies, legal status, discrimination, and economic marginalization. These structural conditions may intensify power imbalances within relationships and restrict survivors’ ability to leave abusive situations, thereby reinforcing patterns of control [24].

The SEM provides a clear and culturally grounded framework for exploring IPV as a lived experience embedded within migration, culture, and structure. Insights generated through this qualitative inquiry will inform future items to develop a culturally sensitive IPV risk assessment instrument by identifying culturally meaningful constructs and language grounded in survivors’ perspectives.

The Social Ecological Model provided the conceptual framework for this study, framing intimate partner violence as shaped by interacting individual, relationship, community, and societal influences (Fig 1). Because first-generation Latino immigrants live with migration and, for many, precarious immigration status, the framework directs particular attention to how these ecological levels interact rather than operate in isolation.

**Fig 1:**
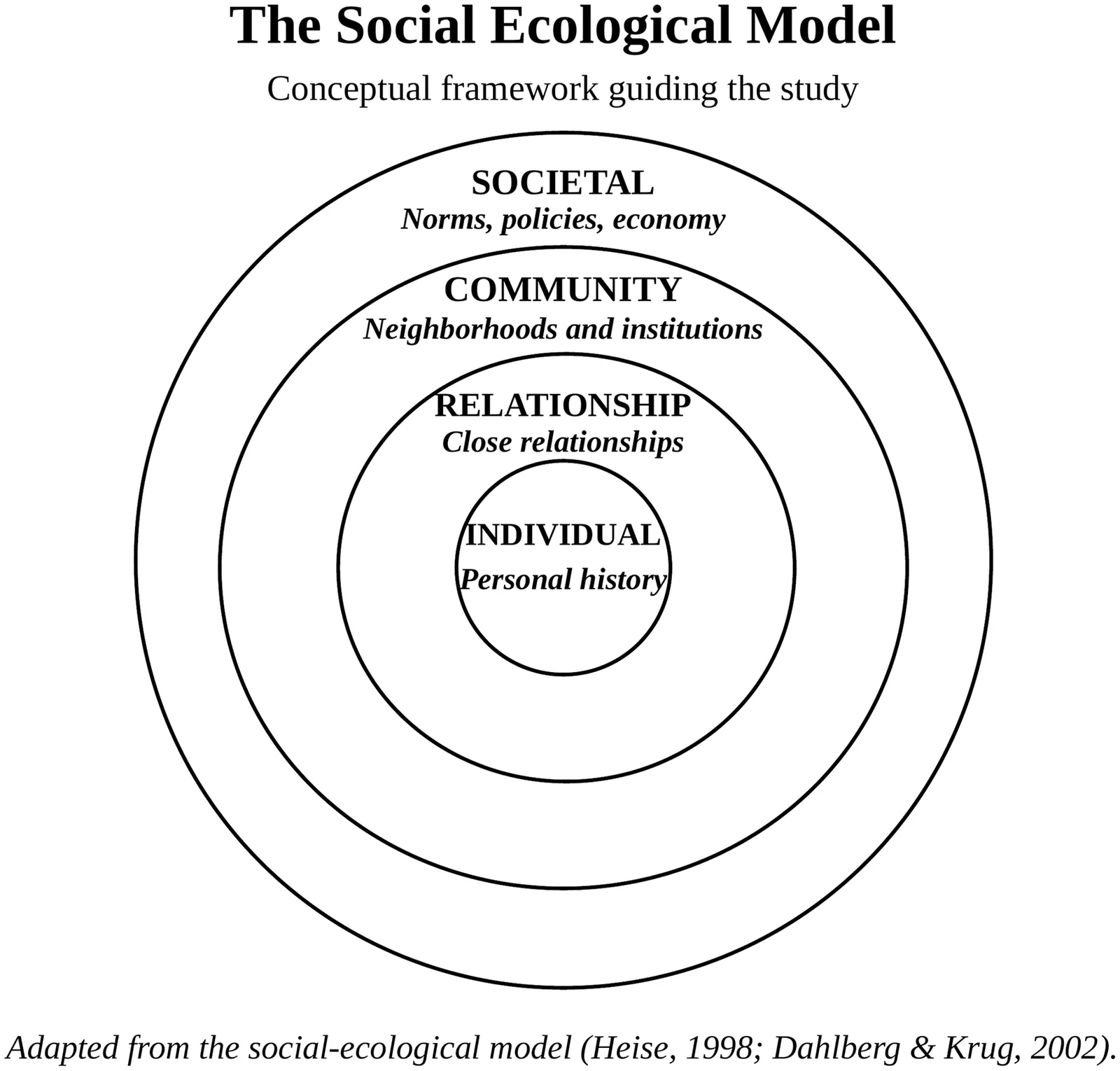
The Social Ecological Model that guided the study, framing intimate partner violence as shaped by interacting influences at the individual, relationship, community, and societal levels. Adapted from the social-ecological model [35,38].

## Materials and methodology

### Study design

This study employed a qualitative phenomenological design to explore and describe the lived experiences of intimate partner violence (IPV) among first-generation Latino immigrants in Southern New Mexico. Phenomenology is appropriate when the aim is to understand how individuals perceive, interpret, and make meaning of a shared phenomenon in their everyday lives [39]. Given the sensitive, deeply personal, and contextually embedded nature of IPV, particularly within immigrant and border-region contexts, this phenomenological approach allowed for rich, in-depth exploration of participants’ experiences, meanings, and coping processes.

### Setting

The study was conducted in Southern New Mexico, a predominantly rural, border-region area with a large Latino population and a substantial number of immigrants. This region is characterized by limited access to specialized healthcare, behavioral health services, and culturally and linguistically responsive social services. These contextual factors are particularly salient for survivors of intimate partner violence (IPV) and were considered essential to understanding participants lived experiences. Participants were recruited through community-based settings, including La Casa, Inc., a domestic violence shelter serving Deming and Las Cruces, as well as community groups located in Anthony, Chaparral, Sunland Park, Las Cruces, and Chamberino.

### Participants and sampling

Participants were eligible for inclusion if they: (a) self-identified as first-generation Latino immigrants; (b) had resided in Southern New Mexico for at least one year; (c) were currently living with or had previously lived with an intimate partner after immigrating to the United States; and (d) were 18 years of age or older and identified as male or female.

Prospective participants were also eligible if they had either personally experienced or observed intimate partner violence; this was ascertained by self-report using a brief study-developed screening survey rather than a validated instrument. This inclusive criterion was adopted deliberately, because requiring individuals to declare personal victimization at recruitment risked suppressing participation, given the shame associated with IPV in this population and heightened concerns about privacy and safety during a period of intensified immigration enforcement.

A combination of convenience and snowball sampling strategies was used. Convenience sampling was initially employed to recruit participants through a domestic violence shelter population. Snowball sampling was subsequently used to extend recruitment into the broader community through trusted community leaders, organizations, and informal networks. Recruitment materials were distributed in both Spanish and English and posted in community locations frequented by Latino residents, including bus centers, public transportation buses, paleterías, mercados, food pantries, and New Mexico State University announcement outlets.

Additional recruitment occurred through community-based organizations, health clinics, and word-of-mouth referrals. Sampling continued until informational adequacy and experiential depth were achieved, consistent with qualitative standards for data sufficiency. Participant recruitment began on March 1, 2025, and ended on May 27, 2025.

### Ethical considerations

This study received approval from the New Mexico State University Institutional Review Board prior to data collection. Given the sensitive nature of intimate partner violence (IPV) and the potential vulnerabilities associated with immigration status, extensive measures were taken to protect participants’ safety, privacy, and confidentiality throughout the research process. All participants provided informed consent prior to participation. The consent process emphasized the voluntary nature of participation, the right to withdraw at any time without penalty, and assurance that participation or nonparticipation would not affect access to services or community support.

The approved protocol number was 2405124955A002, and administrative approval was subsequently granted for an amendment to the study procedures. After the study procedures were explained, participants provided written informed consent, documented on the IRB-approved consent form, prior to the interview.

Interviews were conducted in private and safe locations or through secure communication platforms, depending on participant preference and safety needs. Participants were also provided with information about local IPV-related and supportive resources following the interview. To protect confidentiality, pseudonyms were assigned, and all identifying information was removed from interview transcripts. Audio recordings and transcripts were stored on password-protected, encrypted devices accessible only to the research team.

### Data collection

The study was originally designed to use focus groups. After approximately three months without successful recruitment, attributed in part to the sensitive nature of IPV and participants’ concerns about privacy during a period of heightened immigration enforcement, the team submitted an amendment to the New Mexico State University Institutional Review Board, which was approved under the same protocol, to change the method to individual interviews. Each participant took part in two individual meetings: the first was the in-depth interview, and the second was a follow-up meeting to review and confirm the preliminary findings as a form of member checking. Participants received a $25 gift card after each meeting, for a total of $50.

A total of 24 in-depth, semi-structured individual interviews were conducted by two members of the research team, one English-speaking and one bilingual in English and Spanish. Interviewers used a trauma-informed and culturally responsive approach to foster participant safety, trust, and comfort throughout the interview process. Interviews were conducted in participants’ preferred language (English or Spanish) to support expressive accuracy and reduce language-related barriers to disclosure. All study materials originally developed in English were translated into Spanish and back translated to ensure linguistic and conceptual equivalence.

A semi-structured interview guide with open-ended questions was used to elicit participants’ narratives regarding their experiences of IPV, the meanings they attributed to those experiences, and the personal, relational, cultural, and structural contexts in which those experiences occurred. The semi-structured interview guide was developed based on a literature review, the principal investigator’s prior similar research questionnaire (Ade-Oshifogun et al, 2025), and consultations with experts in the field. The interview guide included questions eliciting participants’ experience of IPV and demographic data. All participants were first asked two open questions about the prevalence of IPV in this population and perceived contributing factors. A set of predetermined, neutrally worded probes was then used to elicit additional details only when a domain had not already arisen spontaneously. Probe wording was fixed in advance and phrased to invite rather than presume experiences (for example, asking whether immigration status “came into play” rather than asserting that it did), and probes were not applied uniformly to all participants. The interview guide covered key areas such as cultural issues, financial instability, domestic violence, and gender role conflicts. Interviews were audio-recorded with participants’ consent and transcribed verbatim for analysis. All interviews were conducted in private rooms with only the participant and researcher(s) present.

The two core questions were deliberately framed at the community level rather than asking participants to disclose personal victimization at the outset. Given the heightened immigration-enforcement climate during data collection and the recruitment difficulties it created, this framing was adopted as a safety-oriented, trauma-informed strategy to reduce the risk of exposure to a vulnerable population and to lower the threshold for participation. Although the two core questions were framed at the community level, participants responded predominantly with first-person accounts of their own experiences of IPV, and the analysis draws on these personal narratives.

Core questions were posed to all participants; the probes were optional follow-ups used only when a topic had not been raised spontaneously. The probes were open-ended and non-suggestive. Coding did not formally distinguish spontaneously raised from probe-elicited content

With participants’ consent, all interviews were audio-recorded. Transcription and initial Spanish-to-English translation were performed using Sonix ai®, an automated transcription and AI-assisted translation program. A bilingual member of the research team then reviewed the translated transcripts against the recordings to verify that meaning, context, and integrity were preserved, and the analysis was conducted on the reviewed English-language transcripts. Although 35 volunteers were available, 24 individuals were interviewed, and data saturation was reached during data collection, as no new concepts emerged in the later interviews. Consistent with the study’s focus on the lived experience of IPV, two participants who described observing rather than personally experiencing IPV were excluded from the analysis, resulting in an analytic sample of 22. Review of the complete audit trail confirmed that this exclusion did not alter the identified themes or the point at which data saturation was reached.

### Data analysis

Data analysis was conducted using thematic analysis informed by phenomenological principles. Analysis began with repeated reading of transcripts to achieve immersion in the data. Initial coding was conducted line-by-line, focusing on significant statements, meanings, and experiential descriptions. Codes were then compared across transcripts and clustered into broader categories and emerging themes. Throughout the analysis, attention was maintained on preserving participants’ voices and meanings while identifying shared patterns across experiences. Analytic memos were written to document interpretive decisions and emerging insights. Themes were refined through iterative comparison with the data until a coherent, and thematic structure was achieved.

Consistent with a phenomenological approach [39], coding was carried out by all members of the research team. Line-by-line coding was performed on each transcript, by each team member; subsequently, the codes were checked again by the team at large.

Coding proceeded over at least four iterative rounds, all documented in the audit trail, and was organized using a codebook in which themes and subthemes were defined using participant-driven, data-grounded definitions. Data was initially organized and analyzed using Intellicus® software, which generated an independent set of candidate codes. Line-by-line manual coding by the research team followed. If the software-generated codes diverged from the team’s coding, the line-by-line coding took precedence. The software therefore supported and cross-checked but did not replace the researchers’ interpretive analysis. The few disagreements that arose were resolved through collective deliberation and consensus, informed by the audit trail and the team’s reflexive journals.

### Methodological rigor

Methodological rigor in this phenomenological study was ensured through the systematic application of strategies designed to enhance credibility, dependability, confirmability, and transferability, consistent with established standards for qualitative research [40]. Given the sensitive nature of intimate partner violence and the sociocultural and immigration-related vulnerabilities of first-generation Latino immigrants in Southern New Mexico, particular attention was paid to ethical, cultural, and relational dimensions of rigor throughout the research process.

*Credibility* was established through prolonged engagement with the phenomenon, in-depth semi-structured interviews, and careful attention to participants’ narratives. Interviews were conducted in participants’ preferred language (English or Spanish) to ensure that participants could express their experiences, emotions, and meanings with minimal linguistic constraint. The interviewer adopted a trauma-informed, culturally responsive approach, allowing participants to guide the depth and pacing of their narratives while ensuring emotional safety. To enhance the authenticity of the findings, member checking was conducted. All 22 participants were invited to review a summary of their interview, and 20 (91%) responded. Seven reviewed the summary by email and the remainder by telephone, during which the same bilingual team member read the summary aloud. All confirmed that the summaries accurately reflected their accounts and required no changes. Peer debriefing was conducted with an experienced qualitative researcher to critically examine emerging codes, themes, and interpretive decisions, thereby strengthening analytic rigor and minimizing interpretive bias.

*Dependability* was supported through the maintenance of a detailed audit trail documenting all phases of the study, including decisions related to recruitment, data collection, interview guide refinement, coding procedures, and theme development. Analytical steps were systematically recorded, and iterative versions of the codebook were preserved to demonstrate the evolution of the analytic process. A consistent analytic approach was applied across all interviews using thematic analysis informed by phenomenological principles.

*Confirmability* was strengthened through reflexive journaling and systematic documentation of analytic decisions. The principal investigator maintained a reflexive journal throughout the study to bracket personal assumptions, professional experiences, and prior knowledge related to intimate partner violence, immigration, and Latino communities. Excerpts from interview transcripts were retained to support all major themes and subthemes, creating a transparent link between data and interpretation. Peer review of coded transcripts and thematic structures further enhanced confirmability.

*Transferability* was supported through the provision of thick, rich descriptions of participants, settings, and contextual conditions. Attention was given to describing the sociocultural, immigration, and geographic context of Southern New Mexico, including border-region dynamics, rurality, and healthcare access challenges that shape the lived experiences of first-generation Latino immigrants. This enables readers to assess applicability to other similar contexts.

*Phenomenological Integrity* – Throughout the study, the analytic focus remained centered on participants lived experiences and meaning-making processes. Data collection and analysis emphasized how participants perceived, interpreted, and navigated intimate partner violence within the context of immigration, culture, family, and structural vulnerability, ensuring alignment with phenomenological inquiry.

## Results

Thematic analysis of the interview data revealed several interconnected themes that illustrate how first-generation Latino immigrants experience and interpret intimate partner violence (IPV) within the broader context of migration, culture, and structural constraints. Participants’ narratives reflected both culturally specific and shared or universal dimensions of IPV.

A total of 22 adults aged 18 years and older comprised the analytic sample (Table 1). As shown in Table 1, the sample was predominantly female (95.5%), and all participants (100%) identified Mexico as their country of origin. Most participants (63.6%) had lived in the United States for 21 years or longer. With respect to marital status, over half of the participants (59.1%) reported being currently married; this category included individuals living with a spouse, living together but emotionally separated, or living separately within the marriage. The remaining participants identified as single/never married, divorced, widowed, or legally separated. At the time of the interviews, all participants resided in the southwestern region of New Mexico, USA, and had lived in the state for at least one year. More than half (59.1%) had resided in New Mexico for over 11 years. As presented in Table 2, the mean age of participants was 52.05 years. Among the 13 participants who were currently married, the mean length of marriage was 25.25 years. Additionally, the mean duration of residence was 19.96 years in New Mexico and 27.45 years in the United States.

**Table 1:** Demographics of Participants (n = 22).

Variable	Category	n (%)
**Age (years)**	18–30	**2 (9.1%)**
	31–40	**1 (4.5%)**
	41–50	**5 (22.7%)**
	51–60	**9 (40.9%)**
	≥61	**5 (22.7%)**
**Gender**	Male	**1 (4.5%)**
	Female	**21 (95.5%)**
**Country of origin**	Mexico	**22 (100.0%)**
**Marital status**	Not married or single	**4 (18.2%)**
	Married (living with spouse)	**10 (45.5%)**
	Married (living together but emotionally separated)	**1 (4.5%)**
	Married (living together but physically separated—different rooms)	**2 (9.1%)**
	Legally separated	**2 (9.1%)**
	Widow/Widower	**0 (0.0%)**
	Divorced	**3 (13.6%)**
**Years of marriage (if currently married; n = 13)**	1–10	**4 (30.8%)**
	11–20	**2 (15.4%)**
	21–30	**2 (15.4%)**
	≥31	**5 (38.5%)**
**Length of time in the USA (years)**	1–10	**6 (27.3%)**
	11–20	**2 (9.1%)**
	21–30	**4 (18.2%)**
	≥31	**10 (45.5%)**
**Length of time in New Mexico (years)**	1–10	**9 (40.9%)**
	11–20	**4 (18.2%)**
	21–30	**2 (9.1%)**
	≥31	**7 (31.8%)**

**Table 2:** Mean and Standard Deviations of Specific Demographic Variables (n = 22).

Variable	M ± SD
**Age (years)**	**52.05 ± 12.40**
**Number of children**	**3.00 ± 1.35**
**Years of marriage (n = 13)**	**25.25 ± 15.14**
**Length of residence in the USA (years)**	**27.45 ± 19.03**
**Length of residence in New Mexico (years)**	**19.96 ± 15.41**

### Themes and subthemes

The major themes identified were Cultural Factors, Abuse, Culture of Silence, Control, Universal Factors, and Systemic Barriers to Legal and Support Resources (Table 3). Within these broader themes, several subthemes further elucidated the nuances of participants’ experiences, including the role of upbringing, normalization of violence, fear, dependency, and barriers to accessing legal and support resources. Together, these themes and subthemes provide a comprehensive understanding of how IPV is experienced, interpreted, and navigated by first-generation Latino immigrants.

**Table 3:** Themes and Subthemes Identified in Participants’ Narratives.

Main Theme	Subthemes
**Cultural Factors**	Machismo; Upbringing and Lack of Education; Normalization of Violence; Language Barrier; Religious Pressure; Early Marriage
**Abuse**	Economics; Intergenerational Abuse; Emotional and Physical Violence
**Culture of Silence**	Shame; Immigration Status; Generalized Fear; fear of Homicide/Death
**Control**	Isolation; Dependency and Entrapment Mechanisms; Manipulation (, Jealousy, Possessiveness, and Accusation)
**Universal Factors Related to IPV**	Children; Abuse Cycle and Patterns; Impact of Trauma; Mental Health
**Systemic Barriers to Legal and Support Resources**	Legal System Challenges; Limited Resources/Financial Barriers; Lack of Awareness and Resources

### Cultural factors

Participants identified one or more cultural factors as having a direct influence on their experiences of intimate partner violence (IPV). In this study, culture refers to the shared beliefs, values, traditions, language, and behavioral expectations held within a community or social group. Participants described these cultural influences as shaping not only their understanding of IPV but also their decisions to remain in or endure abusive relationships. The most prominent cultural factors identified included machismo, upbringing and lack of education, normalization of violence, language barriers, and religious pressure.

### Machismo

Seventeen participants explicitly identified machismo as a significant influence on their IPV experiences. Within many Latin American cultural contexts, machismo reflects the belief that the man holds authority in the household with the woman’s role to serve him. Participants described this as reinforcing rigid gender expectations and placing disproportionate emotional, domestic, and caregiving demands on women. As one participant explained, “*…because of machismo, my partner wants me to be 100 percent in all roles as a woman, to cook, to work, to take care of our daughter, to clean the house, to take care of him, to take care of his emotional needs, everything…*” (Margarita). These expectations often created a cycle of dependency and subordination that made it more difficult for women to leave abusive relationships.

### Upbringing and lack of education

Fifteen participants described their upbringing and limited educational opportunities as important factors that contributed to their vulnerability to IPV and their difficulty leaving abusive partners. These narratives reflected the influence of early family environments, including exposure to violence, gendered socialization, and restricted access to formal education. One participant shared, “*It is because the same goes for the ancestors who had no education, because many of them could not read or write. So, it was like… a little chain, which, well, since that's the way I was raised, I think that's the way it was…*” (Yolanda). Participants’ accounts suggested that these early life experiences shaped beliefs about relationships, conflict, and acceptable behavior, often reinforcing patterns that persisted into adulthood.

### Normalization of violence

Eleven participants discussed the normalization of violence, describing how exposure to violence during childhood contributed to viewing abusive behavior as ordinary or expected within intimate relationships. Participants often linked their current experiences to patterns they had witnessed in their families of origin. As one participant stated, *“…I think it’s like a pattern. If you see your father and mother arguing, it will become an everyday occurrence, something normal, to say, no, well, it’s normal that they fight, that’s what it is…”* (Sandra). These narratives illustrate how repeated exposure to violence in childhood may shape relational expectations and contribute to the intergenerational continuation of abusive dynamics.

### Language barriers

Six participants identified language barriers as an important factor that complicated their experiences of IPV and limited their ability to seek help. Difficulty communicating in English often created barriers when interacting with law enforcement, courts, and community-based services. This hindered participants’ ability to report abuse, explain their situations, and advocate for themselves. One participant recalled, *“…One of the times he arrived, I dialed the police, and the police spoke to me in English, and I did not know what to say… I could not tell them what was going on…*” (Beatriz). These findings suggested that language barriers may intensify survivors’ isolation and reduce access to protection and support.

### Religious pressure

Four participants described religious pressure as a factor that discouraged them from leaving abusive relationships. This pressure was often rooted in traditional Catholic beliefs and cultural teachings that emphasized the permanence of marriage and discouraged divorce. One participant explained, “*I was raised Catholic, a person should not get divorced, especially if you get married in the church. But if you are with a person, you should not leave, you have to be with that person forever, for the rest of your life*.” (Fernanda). For these participants, religious beliefs were not always experienced as sources of comfort or support; rather, they sometimes functioned as constraints that reinforced endurance of abuse.

### Abuse

The theme of abuse reflects participants’ descriptions of the many forms of harm they experienced in intimate relationships. These included economic, intergenerational, physical, and emotional/psychological abuse, which often occurred together rather than separately. Abuse was identified by 21 participants as a central part of their IPV experiences.

### Economic abuse

Seventeen participants described economic abuse, which involved efforts by their partners to control or limit their financial independence. This included withholding money, controlling household spending, preventing employment, or excluding them from financial decisions. One participant stated, “*No, he never gave me money. He never gave me any money. He, he bought everything, even groceries*” (Jessica). These experiences left many women financially dependent and made it harder to leave abusive relationships.

### Intergenerational abuse

Eleven participants described intergenerational abuse, referring to patterns of violence passed down across generations through family experiences and learned behaviors. Participants often connected their current experiences with what they had seen or experienced growing up. One participant reflected, “*Well, I think it is from our childhood, from our home, from our parents that, well, lack of communication and the low values that we have at home, or it comes from parents who are also violent with our parents from generation to generation*” (Teresa). These accounts suggested that early exposure to violence shaped the perception and experience of abuse later in life.

### Physical abuse

Nine participants reported physical abuse, including being hit, pushed, or assaulted with objects. These experiences reflected both direct violence and the constant fear of harm within the relationship. One participant shared, “*He would throw the pots around; if he arrived and the food wasn't ready, he would get angry. I never let the beatings defeat me, but they physically assaulted me*” (Silvia). These accounts highlighted the physical danger and instability participants faced in their homes.

### Emotional and psychological abuse

Nine participants described emotional and psychological abuse as a persistent and harmful part of their experiences. This included threats, intimidation, humiliation, fear, and attempts to control how they felt or saw themselves. One participant explained, “*It was more psychological… if I didn’t leave, he’d talk to immigration; he wasn’t going to give me money; he’d take my car and run me out of the house*” (Sandra). These accounts show how emotional abuse was used to create fear, reduce self-worth, and maintain control.

### Culture of silence

A culture of silence emerged as a strong theme in participants’ experiences of IPV. Many participants described remaining silent about abuse because of shame, immigration-related concerns, generalized fear, and fear of death or homicide. These factors often worked together to prevent disclosure, delay help-seeking, and keep survivors trapped in abusive relationships.

### Shame

Twelve participants described shame as a major reason for remaining silent about their abuse. Participants often expressed concern about how others, especially family and community members, would view them if they disclosed the violence or left the relationship. Shame appeared to be closely tied to social expectations, family reputation, and fear of judgment. One participant reported “*My mom tells me, what would her family say, what would her neighbor say, what would people say, I mean, what would people say….*.” (Rosa)

### Immigration status

Ten participants described immigration-related concerns as an important factor contributing to silence. For some, abusive partners used immigration status as a tool of control by threatening deportation, legal trouble, or separation from family. These threats created fear and uncertainty, making participants less likely to seek outside help. One participant recounted her partner saying “*I am going to turn you in to immigration so they can take you to Mexico, either they will take you or they will put you in jail because you are an immigrant*.” (Jessica)

### Generalized fear

Eight participants described fear as a major reason they stayed silent. This fear included fear of retaliation, fear of what might happen if they spoke out, and fear of the unknown if they attempted to leave. Participants often described fear as something constant that shaped their daily decisions and prevented them from reaching out for support. As Jessica stated, r “*it’s fear, that’s totally fear that we women have*.”

### Fear of death or homicide

Four participants described the most extreme form of silence as being driven by fear of death or homicide. These participants reported direct threats to their lives and, in some cases, threats involving their loved ones. Such threats created intense fear and reinforced silence as a survival strategy. This fear is real in one of the participants’ descriptions below:

*“And then he started to harass me … and threaten me… that … if he realized that I was going to separate, he was going to kill me, he was going to tear me to pieces. He told me, “You know that I am capable of doing that to you. And I knew that he was capable, I was trembling with fear, and even now I say, oh my God, how scary is that? I mean, how can they corner you like that?”* (Monica)

### Control

The theme of control reflects participants’ experiences of behaviors used to dominate, restrict, and limit their independence. Sixteen participants identified control as a key part of their IPV experiences. These experiences were grouped into five related subthemes: isolation, dependency and entrapment, manipulation, jealousy and accusation, and possessiveness.

### Isolation

Eleven participants described isolation as a strategy used by their partners to limit contact with family, friends, and other support systems. This increased dependence on the abuser and reduced opportunities to seek help. One participant explained, “*If I wanted to go to the restroom, I would have to ask permission… what are you doing over there?*” (Isabel). These experiences demonstrated how constant monitoring and restriction of movement reduced personal freedom and reinforced control.

### Dependency and entrapment

Eight participants described dependency and entrapment, often through financial control or interference with employment. These actions made it difficult to leave the relationship. One participant shared, “*I couldn't ask for a job because… he would give out the wrong information every time”* (Isabel). Such experiences limited financial independence and trapped participants in abusive situations.

### Manipulation

Six participants described manipulation as a control tactic used by their partners. This included limiting their ability to work, make decisions, or act independently. One participant stated, “*He wouldn't let me work… you can’t do anything without me he would say*” (Monica). These behaviors reduced confidence and reinforced dependence.

### Jealousy and accusation

Six participants described jealousy and accusations of infidelity as common forms of control. These behaviors often led to conflict and, in some cases, violence. One participant noted, “*Jealousy brings a lot of violence*” (Rosa). These accounts suggest that jealousy was often used to justify controlling behaviors.

### Possessiveness

Six participants described possessiveness as a form of control that limited their independence and freedom. One participant explained, “*They take away our independence and forfeit our freedom*” (Rosa). These behaviors reinforced dependence and restricted personal autonomy.

### Universal factors related to IPV

Several universal factors of IPV emerged in this study, reflecting experiences that are commonly reported across different populations and cultural groups. These included the cycle of abuse, the role of children, mental health concerns, and trauma. Although participants’ experiences were shaped by cultural and immigration-related factors, these themes also reflected broader patterns commonly seen in IPV research.

### Cycle of abuse

Six participants described the cycle of abuse as a recurring and difficult-to-break pattern that often extends across generations. Participants explained that abuse may continue when it is normalized, hidden, or never addressed. One participant shared, *“…because a person follows the cycle of the family, if someone doesn’t report it, then the cycle keeps going to not report*” (Estela). These accounts suggest that silence, learned behavior, and repeated exposure to violence can reinforce the continuation of abusive relationships across time.

### Children

Six participants described children as an important influence in their decisions to either remain in or leave abusive relationships. For some, having children increased fear and led them to stay in the relationship in an effort to protect or provide for them. One participant stated, “*Many women keep quiet, yes they do, they do keep quiet out of fear, because they have children”* (Julia). For others, children became the main reason to leave, especially when participants feared that the abuse would continue or affect the next generation. One participant explained, “*What really motivated me was… I had my son. And I didn't want my son to go through all what I had*” (Isabela). These findings illustrated that children may act as both a reason for silence and a motivation for change.

### Mental health concerns

Six participants described experiencing mental health difficulties while enduring IPV. These included feelings of depression, sadness, emotional exhaustion, hopelessness, and low self-worth. Participants often described how prolonged abuse affected their identity, confidence, and sense of self. One participant explained, “*It got to a point I didn’t feel like me anymore… where he put me into depression… I was feeling so ugly… I would say, this happens to me because I deserve it, that is, you justify the abuse because you already feel so bad about yourself*” (Julia). These narratives demonstrated how IPV can deeply affect emotional well-being and lead survivors to internalize blame for the abuse.

### Trauma

Trauma was another common and deeply rooted experience among participants. Several women described carrying the emotional and psychological effects of IPV long after the abuse occurred. One participant reflected, “*I’m older and I see the big picture because I tell you there’s traumas that we carry and carry and carry*” (Patricia). These findings suggested that the effects of IPV often extend beyond the immediate abusive experience and may continue to affect survivors’ emotional health, relationships, and daily functioning over time.

### Systemic barriers

Participants described systemic barriers as external conditions that made it harder to access protection, services, and support. These barriers included lack of awareness of available resources, legal system challenges, and financial hardship. Together, these limitations increased dependence on abusive partners and often delayed or prevented help-seeking.

### Lack of awareness of resources

Several participants described being unaware of the services and support available to them. For many, help was not sought because they simply did not know where to go or what options existed. One participant explained, “*I think that there are many people who are… how do you say the word?… who do not know about all the help that is out there now*.” Another participant shared, “*It wasn't until I suffered the violence that I started to look for help for myself. I went to an association in El Paso, and they started to direct me to places where they can help me with school, with diapers, with things like that, that I didn't know existed*.” These accounts suggested that many survivors may remain isolated or unsupported until the abuse becomes severe enough to force them to seek help.

### Legal system challenges

Participants also described major difficulties with the legal system, including fear of deportation, limited understanding of legal rights, and gaps in legal protection across state or local jurisdictions. These barriers often discouraged survivors from reporting abuse or seeking legal support. One participant recalled, “*They only took pictures. And, unfortunately, they said that because it happened in Texas and I live in New Mexico, there was nothing to be done. He only took the pictures. I had a black eye and bruises on my hands*.” Despite visible signs of abuse, no further action was taken. Such experiences reflected how legal gaps, fear, and mistrust of systems may leave survivors feeling unprotected and unsupported.

### Limited financial resources

Limited financial resources were another major barrier to safety. Participants described how poverty, unstable income, and economic dependence on their partners made it difficult to leave abusive relationships or care for their children independently. One participant explained,

“*They don't consider the rent, they don't consider the car payment... the woman who has the child most of the time is more enslaved... And they don't even make the father give a percentage of his salary. I mean, it's not like that, it's not like that; it's very, very little what they take from him for child support... because the more she grows, the more expenses she has*. *She needs clothes and shoes and so on.”*

This account exhibited how financial strain can leave survivors feeling trapped and overwhelmed, especially when systems meant to provide support are inadequate.

## Discussion

Participants’ accounts align with evidence that traditional gender norms and power imbalances shape IPV in Latino communities [24,25], but they also qualify it: machismo mattered here less as a fixed cultural trait than as an expectation that was reasserted after migration, which helps explain why it surfaced so strongly in this sample. Reports of childhood exposure to violence are consistent with intergenerational transmission described elsewhere [41], and the role of language barriers and religious pressure in discouraging help-seeking mirrors prior findings on immigrant Latina survivors [24,42]. Early marriage, by contrast, was less central than in studies that foreground it [43], suggesting its relevance in this population is indirect, operating through dependence rather than as a primary driver.

The prominence of economic abuse converges with work identifying it as a principal barrier to leaving for immigrant women [10,42], and the co-occurrence of physical, emotional, and economic abuse fits evidence that IPV rarely involves a single form [2]. Where our findings extend the literature is in showing how tightly these forms were bound to immigration-related vulnerability: emotional and psychological abuse was intensified by fear and isolation specific to the migration context [37,42], suggesting that for this population the forms of abuse are not merely overlapping but mutually reinforcing.

Consistent with prior work, shame functioned as a central barrier to disclosure [20,44]. Our findings diverge, however, from accounts that treat silence primarily as an individual coping response: here silence was actively produced by external threats, as partners weaponized deportation and control of documents [19,20], and generalized and homicide-related fear delayed help-seeking, sometimes until the danger became severe [19,26,45,46]. The study therefore reframes silence in this population as an externally enforced condition rather than a personal disposition.

The control tactics participants described match established models of coercive control, including isolation, dependency, manipulation, and possessiveness [2,36,37]. What differs is their texture in an immigrant context: isolation extended beyond social separation to language and institutional exclusion, and dependency was amplified by restricted employment and documentation [10,47]. These findings suggest that generic coercive-control frameworks may understate how structural conditions convert ordinary control tactics into near-inescapable entrapment for immigrant survivors.

Several dynamics converged clearly with the broader IPV literature and are best read as confirmation rather than novelty: the cycle of abuse [48], children as both a reason to stay and a reason to leave [13,44], and the mental-health and trauma consequences reported among Hispanic and other survivors [49,50]. Their value here is to show that culturally specific pathways did not replace these universal mechanisms but operated alongside them, which cautions against framing immigrant IPV as categorically distinct from IPV in other populations.

Finally, the systemic barriers participants faced are congruent with evidence that immigrant survivors encounter limited service awareness, legal-system mistrust, and economic constraint [21,24]. The study adds specificity by showing these barriers acting together rather than in isolation: fear of deportation, thin culturally responsive services, and financial dependence compounded one another, so that any single intervention was unlikely to open a safe route out. This points, more directly than much of the existing literature, to the need for structural rather than individual-level responses for this population. These findings together do more than align with or diverge from prior work; they reveal how the ecological levels interact in this population, which the following section develops by extending the Social Ecological Model.

### Extending the social ecological model

The six themes point to a single generative condition rather than a set of independent risk factors. For first-generation Latino immigrants, migration destabilized established gender roles and economic arrangements while it simultaneously removed the community and legal buffers that may constrain abusive behavior [19,24]. This helps explain why the themes emerged in the specific configuration observed here. Machismo did not merely persist after migration; it was reasserted as men sought to recover authority lost through economic dependence, unfamiliar surroundings, and shifting household roles, while women were expected to absorb this reassertion as endurance and service. Economic abuse was prominent when resettlement concentrated financial control in the partner with greater language proficiency, documentation, or employment. The culture of silence deepened because the usual avenues for disclosure, whether family, faith community, or the legal system, were themselves sources of stigma or danger in the migration context. In this study, the themes are not parallel descriptions of experience but connected expressions of role renegotiation occurring under conditions of structural vulnerability.

These findings both confirm and extend prior qualitative work on IPV among Latina and immigrant survivors. Earlier studies have documented the influence of traditional gender norms, relational power imbalances, and structural barriers to help-seeking, but have often examined these as separate domains considered one at a time [19,24,25]. The present study contributes an account of how these domains operate together within a single population and, importantly, how they reinforce one another. For example, prior evidence describes fear of deportation and mistrust of authorities as barriers to formal help-seeking [24]. Participants in this study described the same fear but showed that it did more than deter help-seeking; it was actively used by partners as a tool of coercion. This moves the literature from cataloguing discrete barriers toward explaining the relational mechanisms through which structural conditions are converted into interpersonal control.

The most significant theoretical contribution of this study is a refinement of how the Social Ecological Model applies to immigrant survivors. In its conventional form, the model arranges individual, relationship, community, and societal influences as nested but relatively distinct layers, with the outermost societal layer exerting a diffuse background influence [19,35]. Our findings suggest that, for first-generation Latino immigrants, the societal layer does not remain in the background. Precarious immigration and legal status, an outer level structural condition, was drawn inward and enacted at the relationship level as a specific control tactic, namely the threat of deportation or of reporting to immigration authorities and was then internalized at the individual level as fear, shame, and self-silencing. In other words, a single structural condition penetrated and reorganized every inner level of the model. We describe this pattern as cross-level coupling, meaning that the levels of ecology were not merely co-present but were bound together by immigration status acting as a vertical mechanism. Fig 2 presents a migration-sensitive adaptation of the Social Ecological Model that depicts this coupling.

**Fig 2:**
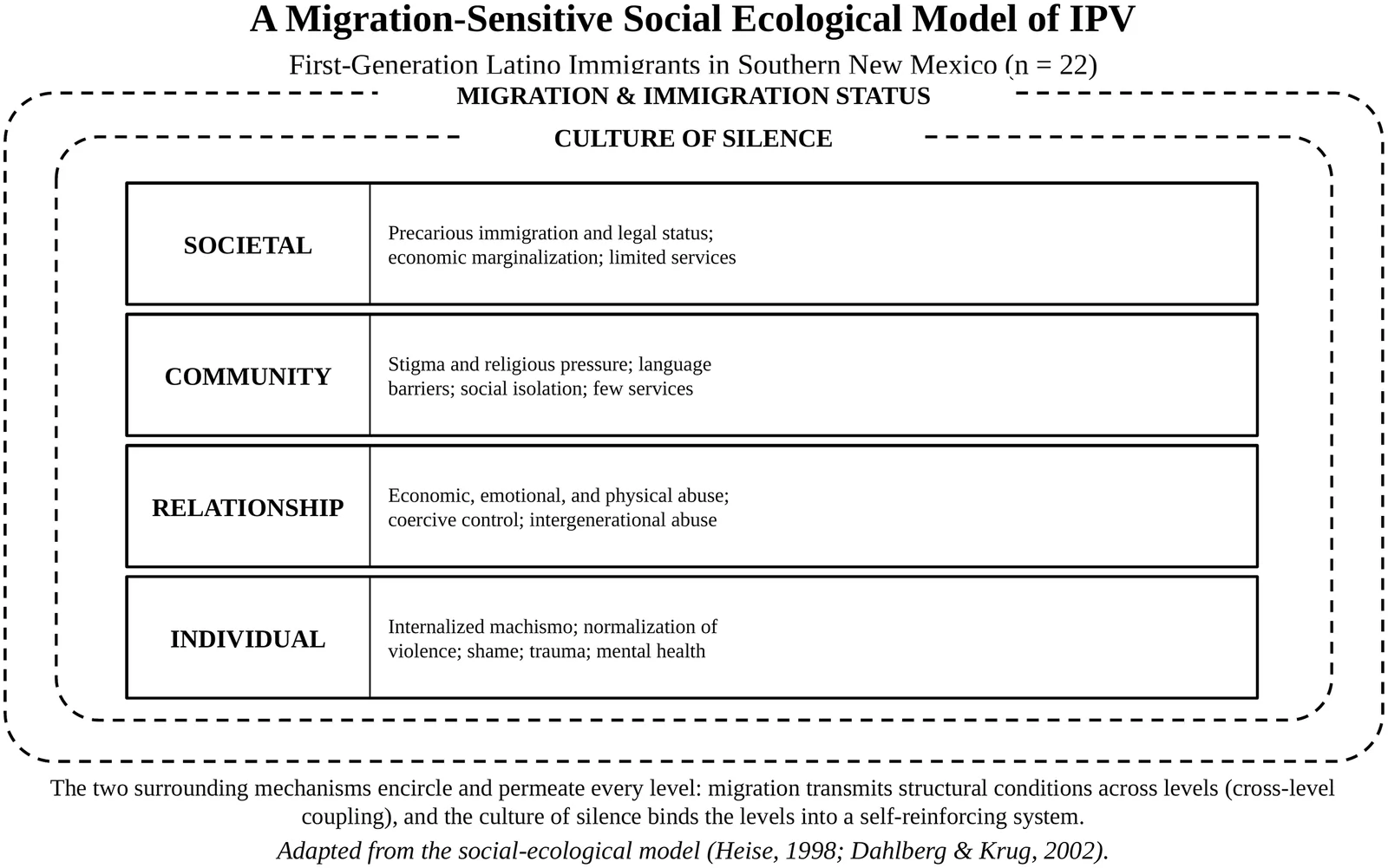
A migration-sensitive adaptation of the Social Ecological Model of intimate partner violence among first-generation Latino immigrants in Southern New Mexico. The four ecological levels are shown as nested tiers with their associated themes. Two surrounding mechanisms, migration with precarious immigration status and the culture of silence, encircle and permeate every level; migration transmits structural conditions across levels (cross-level coupling), and the culture of silence binds the levels into a self-reinforcing system. Adapted from the social-ecological model [35,38].

A second extension concerns the culture of silence. Silence is often interpreted either as an individual coping response or as a community level product of stigma. The narratives in this study indicate that silence operated at all four ecological levels at once and functioned as the connection linking them. At the individual level it appeared as shame and internalized blame; at the relationship level it took the form of secrecy and enforced isolation; at the community level it was sustained by stigma, religious expectation, and the absence of culturally responsive services; and at the societal level it was reinforced by fear of legal and immigration consequences. Rather than being a single outcome located at one level, silence is better understood as a mechanism that couples the levels together and allows abuse to remain both entrenched and invisible. This reconceptualization has direct implications for assessment, because it suggests that silence itself, together with its multiple sources, is a measurable dimension of risk rather than only an obstacle to detection.

The coexistence of culturally specific and universal themes offers a third insight. The universal dynamics reported here, including the cycle of abuse, the influence of children on staying or leaving, trauma, and mental health consequences, are well established across IPV populations [13,41,44,48–50]. What this study adds is evidence that culturally specific scripts and universal dynamics are interwoven rather than parallel. Cultural expectations shaped the meaning participants assigned to abuse and raised the threshold at which it was recognized as abuse, while the universal mechanisms supplied the behavioral patterns through which the abuse was carried out and sustained. Interpreting culture and universality as interacting, rather than as competing explanations, clarifies why interventions that address only one level are likely to be insufficient for this population.

Altogether, these interpretations articulate what is genuinely new in this study. Beyond describing that IPV among first-generation Latino immigrants is shaped by culture, relationships, and structure, the study explains how a structural condition is transmitted across ecological levels through identifiable relational mechanisms, and how silence binds those levels into a self-reinforcing system. This understanding reframes the task of risk assessment. A culturally grounded instrument for this population should not treat individual, relational, and structural risks as independent checklists, but should capture cross-level items such as the use of immigration status as coercion and the multiple sources of silence. In this way the analytic contribution of the present qualitative study provides the conceptual foundation for the culturally sensitive items to be generated in the planned subsequent phase of this research.

### Conflicting evidence and the immigrant paradox

The preceding discussion showed that these findings largely converge with existing evidence. One strand of literature, however, points in the opposite direction and bears directly on how much weight should be placed on culture as a driver of IPV. Because machismo, marianismo, and the culture of silence were prominent in participants’ accounts, one might infer that stronger adherence to traditional cultural values increases exposure to IPV.

Quantitative evidence complicates this inference. In a national sample of Latina women, a Latino cultural orientation was associated with decreased odds of all forms of IPV, whereas a more acculturated, Anglo orientation was associated with increased odds, and immigrants were less likely than United States-born Latinas to report IPV [51]. A meta-analysis of acculturation and IPV likewise found that higher acculturation was associated with greater IPV victimization among Latinas [52]. These two study results describe an immigrant paradox that runs counter to the assumption that traditional culture is the principal driver of IPV, and other studies report the opposite direction or no independent effect of traditional gender roles, indicating that the evidence base is genuinely mixed.

These divergent findings can be reconciled by distinguishing the incidence of IPV from the ways it is experienced, endured, and disclosed. A Latino cultural orientation may be protective against the occurrence of IPV, a pattern also reported in national data on cultural maintenance [53], while at the same time shaping the meaning survivors assign to abuse, the threshold at which they name it, and their readiness to seek help. Marianismo, for instance, has been linked less to the incidence of IPV than to remaining in abusive relationships and to greater psychological distress [18], and the influence of acculturation is gendered and entangled with structural conditions such as economic instability and immigration status [25,37]. Interpreted through the Social Ecological Model, culture therefore does not act as a single individual-level risk factor but operates across levels and through competing pathways, some reducing incidence while others deepen silence, entrapment, and barriers to help-seeking. Seen in this light, the conflicting evidence reinforces rather than weakens the study’s central argument that IPV among first-generation Latino immigrants arises from the interaction of cultural scripts with relational control and structural vulnerability. Rather than undermining the study's findings, this paradox underscores why intimate partner violence in this population must be understood as the product of interacting cultural, relational, and structural forces rather than culture alone.

### Summary of study

The findings of this qualitative study show that intimate partner violence (IPV) among first-generation Latino immigrants is shaped by cultural beliefs, relationship dynamics, overlapping forms of abuse, and systemic barriers. Cultural influences such as gender roles, family expectations, and silence around abuse affected how violence was understood and endured. Participants also described economic, emotional, physical, and intergenerational abuse, along with control tactics such as isolation, dependency, and manipulation that limited their ability to leave. Participants’ experiences also reflected universal aspects of IPV, including the role of children, cycles of abuse, trauma, and mental health effects. At the same time, systemic barriers such as immigration-related fears, financial hardship, and limited access to services made help-seeking more difficult.

Our findings highlight the need for an IPV risk assessment questionnaire that reflects the cultural and structural realities of first-generation Latino immigrants. This would be a culturally relevant tool to improve early identification and support more effective and responsive care.

### Study implications

The findings of this study have important implications for practice, policy, and research.

For practice, the results highlight the need for culturally responsive and trauma-informed approaches to IPV screening and intervention. Healthcare providers, nurses, social workers, and mental health professionals should be trained to recognize non-physical forms of abuse, such as economic abuse, coercive control, and immigration-related threats, which are often missed by traditional screening tools. Building trust, ensuring confidentiality, and providing language-appropriate services are essential to improving engagement, safety planning, and support for survivors.

Through a policy lens, the findings emphasize the need for immigration-informed protection and improved access to legal and social services. Policies should support culturally and linguistically appropriate services, strengthen protection regardless of immigration status, and promote collaboration across healthcare, legal, and community systems. Reducing systemic barriers may help decrease fear and improve access to care and protection.

From a research perspective, this study highlights the need for culturally grounded assessment tools that reflect the lived experiences of Latino immigrant survivors. The identified themes provide a strong foundation for developing a culturally relevant IPV risk assessment questionnaire tailored to first-generation Latino immigrants. Future research should test and validate this tool across different settings and populations, including diverse gender identities, to improve its applicability and usefulness.

### Strengths and limitations

This study has several strengths. Qualitative design allowed for a deep understanding of survivors’ lived experiences, capturing cultural and contextual factors often missed in quantitative studies. Focusing on first-generation Latino immigrants in Southern New Mexico addresses an important gap in the literature and increases the relevance of the findings for immigrant and border communities. The identification of both culturally specific and universal themes strengthens the credibility of the results.

Several limitations should be noted. The use of purposive sampling and a qualitative design limit the generalizability of the findings. Although participants were open to sharing their experiences, their responses may have been influenced by fear, stigma, and recall bias, especially because data collection took place during a period of heightened immigration raids and enforcement activity in the United States. In addition, the sample consisted primarily of female participants, with only one male participant, limiting the representation of male survivors and individuals of other gender identities. Despite these limitations, the study provides rich and meaningful insights, particularly given that IPV disproportionately affects women in this population.

Recruitment relied largely on convenience and snowball sampling. Only one participant was recruited from a shelter; the remaining participants were reached through community meetings and subsequent referrals from participants who knew others with similar experiences. This approach may over-represent individuals connected to community networks and may under-represent those who never sought any form of help or support, and participants’ accounts may or may not have involved formal help-seeking.

In addition, the current study included only one male participant, reflecting the difficulty of engaging male survivors of IPV in this population, where machismo and shame strongly discourage disclosure by men.

## Conclusion

This study indicates that IPV among first-generation Latino immigrants is shaped by a combination of cultural norms, power dynamics, trauma, and systemic barriers. Participants’ experiences highlight how culture, silence, control, and structural challenges work together to sustain abuse and limit help-seeking. These findings underscore the need for culturally responsive, trauma-informed, and immigration-aware approaches to IPV prevention, assessment, and intervention. Importantly, the results provide a strong foundation for the development of a culturally grounded IPV risk assessment questionnaire that reflects the unique experiences of Latino immigrant survivors. Such tools can improve early identification, guide intervention, and support better outcomes for this population.

Consistent with this scope, the present study is limited to reporting the qualitative findings. The development of the instrument, including the specification of a tool framework, the mapping of these themes to questionnaire domains, and item generation and validation, was not undertaken here and represents the planned next phase of this research.
